# Transforming Growth Factor-β1 Drives Neutrophil Chemokinesis, Not Chemotaxis

**DOI:** 10.2147/JIR.S621030

**Published:** 2026-09-30

**Authors:** Louise E Crowley, Ellen Jenkins, Kirsty C McGee, Theodore S Smith, Jon Hazeldine, Robert A Stockley, David R Thickett, Dhruv Parekh, Aaron Scott

**Affiliations:** 1 Department of Inflammation and Ageing, School of Infection, Inflammation and Immunology, College of Medicine and Health, University of Birmingham, Birmingham, UK; 2 University Hospitals Birmingham NHS Foundation Trust, Birmingham, UK; 3 National Institute for Health and Care Research (NIHR) Birmingham Biomedical Research Centre, Birmingham, UK

**Keywords:** chemokines, cytokines, lung, fibrosis

## Abstract

**Purpose:**

Idiopathic pulmonary fibrosis (IPF) is a devastating condition that is characterized by dysregulated repair responses to alveolar epithelial damage and progressive remodeling of the lung interstitium. Neutrophils are critical responders to tissue injury, but their role in organ fibrosis pathogenesis remains unclear. Neutrophil numbers are increased in bronchoalveolar spaces of IPF patients, and neutrophil activation markers are elevated in fibrotic lung tissue. However, the key mediators driving neutrophil movement in fibrotic lung diseases are unknown. Transforming growth factor–beta 1 (TGFβ1) is a critical pro-fibrotic cytokine, but its effect on neutrophil movement remains unclear. This mechanistic, exploratory study aimed to determine whether TGFβ1 alters neutrophil migration in a direction-dependent (chemotaxis) or direction-independent (chemokinesis) manner and to assess whether TGFβ1 exposure is accompanied by functional changes consistent with neutrophil priming.

**Methods:**

This study utilized human neutrophils from healthy donors to model aspects of a TGFβ1-rich environment. Neutrophil movement was assessed using a transwell migration assay and by real-time tracking of human neutrophils within an Insall chamber. Extracellular release of superoxide anions and nucleic acid were assessed by lucigenin-amplified chemiluminescence, and fluorometry, respectively. Expression of cell surface markers, TGFβ receptor 1 and pSmad2/3 were assessed using flow cytometry and Western blot.

**Results:**

This study revealed that TGFβ1 drives neutrophil chemokinesis, but not chemotaxis, and that pre-treatment with TGFβ1 amplified selected movement responses to the established chemoattractants, interleukin-8 and N-Formylmethionine-leucyl-phenylalanine. TGFβ1 also increased total superoxide anion production and nucleic acid release, but did not alter cell surface expression of adhesion (CD11b and CD62L) or degranulation (CD66b) markers.

**Conclusion:**

Together, these data suggest that TGFβ1 promotes neutrophil chemokinesis, rather than chemotaxis, and that this may reflect transition into a priming-like state with potential relevance to neutrophil behavior in conditions such as fibrotic lung disease.

## Introduction

Idiopathic pulmonary fibrosis (IPF) is characterized by progressive deposition of extracellular matrix in the lung interstitium; precipitated by dysregulated repair responses to alveolar epithelial damage. Current anti-fibrotic therapies only slow physiological disease progression, with the mean survival time of patients with IPF remaining less than 5 years.^1^ Novel therapeutic targets are therefore required to improve patient outcomes.

Neutrophils are the most common immune cell in the body and critical responders to tissue injury but they also house an array of mediators that can be deleterious.^2^,^3^ There is growing interest in the role neutrophils may play in organ fibrosis pathogenesis.^4^ Retrospective studies have reported positive associations between neutrophil numbers/degranulation markers in IPF bronchoalveolar lavage fluid and mortality and extent of lung fibrosis.^5–7^ In addition, increased neutrophil activation markers, such as neutrophil extracellular traps (NETs) and neutrophil elastase have been observed in fibrotic lung tissue,^8^,^9^ which may increase tissue damage. Furthermore, inhibition of neutrophil elastase reduces bleomycin induced pulmonary fibrosis in animal models,^10^ suggesting a role in disease pathogenesis. However, the factors driving neutrophil recruitment to sites of organ fibrosis are currently unclear.

The pleiotropic cytokine, transforming growth factor–beta 1 (TGFβ1), is elevated in the circulation and lungs of IPF patients and has well established pro-fibrotic effects on epithelial and mesenchymal cells.^11–16^ TGFβ1 has also been suggested to be a mediator of neutrophil mobilization^17^ although this is controversial.^18–21^ The use of in vitro models of neutrophil movement may explain these inconsistencies by failure to distinguish between direction dependent movement (chemotaxis) and random movement (chemokinesis).^22^ Chemotaxis is a forward movement driven by actin polymerization and pseudopod projection towards a chemoattractant (for example IL-8 and N-Formylmethionine-leucyl-phenylalanine (fMLP))^23^,^24^ whereas during chemokinesis, cytoplasmic extension/retraction is random leading to increased but non-directed movement.^25^

It is therefore important to study the effect of TGFβ1, on these two forms of movement. Inhibition of TGFβ1 may abrogate fibrosis but also impair neutrophil migration into and within an organ thereby reducing any contribution to repair or damage within the fibrotic stroma.^4^

TGFβ1 has been noted by some groups to increase neutrophil respiratory burst and phagocytosis,^26^ whilst others have reported that TGFβ1 either suppresses or has negligible effects on reactive oxygen species production and degranulation.^19^,^21^,^27–30^ TGFβ signaling plays a key role in other physiological and pathological processes throughout the body, such as wound healing, immune homeostasis and tumorigenesis.^31^ Therefore, it is important that the impact of a TGFβ1 rich microenvironment on neutrophil responses is determined as this could have implications in the design and development of new therapeutic strategies in a range of diseases.

The aim of this study was to model the effects of the pro-fibrotic stimulus, TGFβ1, on healthy human neutrophils in vitro. In particular, to assess whether TGFβ1 alters neutrophil migration in a direction-dependent or direction-independent manner and to assess whether TGFβ1 exposure is accompanied by functional changes consistent with neutrophil priming.

## Material and Methods

### Participants

This study was approved by the West Midlands – South Birmingham Research Ethics Committee (REC Number 10/H1208/48). This study complies with the Declaration of Helsinki. All participants provided written informed consent and self-reported as healthy (particularly with no recent or concurrent infection, Supplementary Method).

### Neutrophil Isolation

Peripheral blood neutrophils were isolated from whole blood using dextran (Sigma-Aldrich) sedimentation and discontinuous Percoll^®^ (Cytiva) density gradient centrifugation, as described previously.^32^ After isolation, neutrophils were resuspended at a concentration of 10^6^ to 10^7^ mL^−1^ in RPMI containing 2mM L-glutamine (Gibco), supplemented with 1% penicillin- streptomycin (Sigma-Aldrich) (described as assay media) or HBSS (Gibco^TM^) containing magnesium chloride and calcium chloride (HBSS^+/+^). Neutrophil purity was typically ≥ 95%.

### TGFβ1 Reconstitution

Recombinant human TGFβ1 (R&D Systems, 240-B/CF) was reconstituted in 0.1% BSA (Sigma-Aldrich) – Dulbecco’s Phosphate Buffered Saline (DPBS) (Gibco^TM^) supplemented with 4 mM hydrochloric acid (Fisher Chemical^TM^), denoted as “vehicle” in this study.

### Transwell Migration Assay

Three μm pore size Nunc^TM^ (ThermoFisher Scientific) polycarbonate cell culture inserts were added to a 24-well plate that contained assay media supplemented with TGFβ1 (0.001–10 ng/ mL) or vehicle in the lower compartment. Neutrophils (1 x 10^6^ in assay media) were pipetted onto the transwell inserts and incubated for 90 minutes at 37° C/5% CO_2_. Post-incubation, the transwell inserts were discarded and the contents of each well were collected. Cells were fixed for 10 minutes in 2% paraformaldehyde (ThermoScientific), washed and resuspended in 200 µL 1% BSA-DPBS. A 100 µL sample volume was then counted by flow cytometry using the MACSQuant^®^ Analyzer 10 (Miltenyi Biotec) and data analyzed using FlowJo_v10.9.0 Software. The value counted was multiplied by 2 to determine the total number of migrated cells within each 200 µL sample.

### Insall Chamber Assay

Whilst the transwell assay was used as an initial measure of neutrophil migration, the Insall chamber assay was employed to distinguish direction-dependent chemotaxis from direction-independent chemokinesis. The Insall chamber assay was performed using a modified Dunn chamber (see graphical illustration in Figure 1).^33^

**Figure 1:**
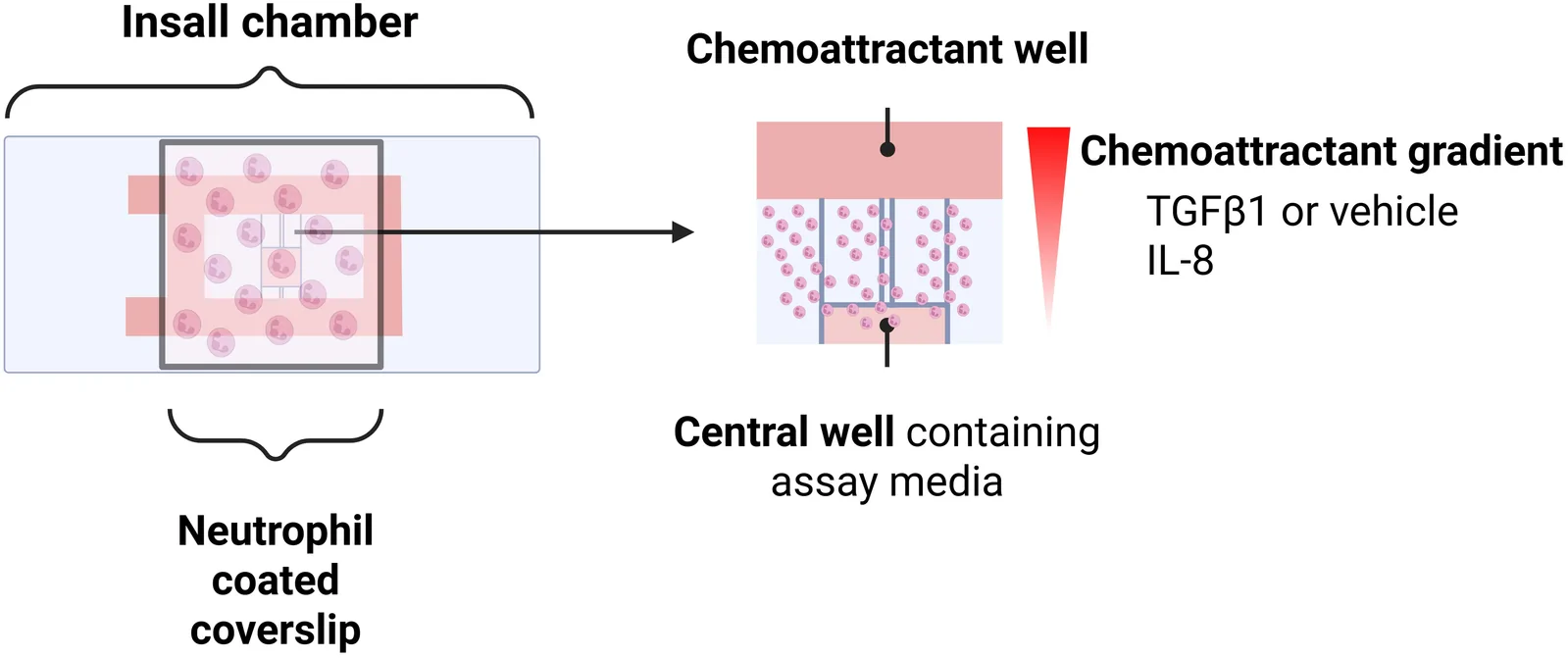
Graphical illustration of Insall chamber assay. Created with *BioRender.com*.

Neutrophils (8 x 10^5^ in assay media supplemented with 0.01% BSA) were added to a 7.5% BSA (Gibco) coated glass coverslip and incubated for 20 minutes at room temperature (RT) to allow adhesion. The neutrophil coated coverslip was then inverted onto the Insall chamber. To create a chemoattractant gradient within the chamber, the chemoattractant well was gently emptied using filter paper and refilled with assay media supplemented with TGFβ1 (0.1–30 ng/ mL), vehicle, IL-8 (100 nM; R&D Systems: 208-IL/CF) or fMLP (10 nM; Sigma: F3506). Images of the chamber slide were captured by a Leica DMI6000 B video capture microscope every 20 seconds for a total of 12 minutes. Images were analyzed using FIJI Image J (National Institutes of Health, USA); 10 randomly selected neutrophils were tracked on each slide using the Manual Tracking plug-in. Supplementary Table 1 defines the parameters used to assess direction dependent (chemotaxis) and direction independent (chemokinesis) neutrophil movement as described previously^22^,^34^,^35^ ensuring post hoc adjustment for minor background slide movement.

### Measurement of Superoxide Anion Release as Indicative of Cell Priming/Activation

Extracellular superoxide anion release was assessed by lucigenin-amplified chemiluminescence. Neutrophils were treated for 15 minutes in assay media supplemented with TGFβ1 (0.1–10 ng/ mL) or vehicle. Following stimulation, neutrophils were centrifuged (1500 x g, 2 min) and resuspended in HBSS^+/+^. Neutrophils (1 x 10^5^) were then pipetted into wells of a 96-well white-bottomed flat plate (Corning^®^) containing 200 µM lucigenin (N, N′-Dimethyl-9,9′-biacridinium dinitrate (Sigma-Aldrich). Extracellular superoxide anion release was measured at 1-minute intervals for 200 minutes using a Berthold Centro LB 960 luminometer (Berthold Technologies, UK). The area under the curve (AUC), representative of total extracellular superoxide anion release, was calculated using GraphPad Prism^®^ (GraphPad Software Ltd, San Diego, CA, USA).

### Flow Cytometric Analysis of Cell Surface Markers as Indicative of Cell Priming/Activation

Surface expression of neutrophil priming/activation markers (CD66b, CD11b and CD62L) were measured on freshly isolated peripheral blood neutrophils as described in the Supplementary Methods. Antibodies used were anti-CD66b-APC (BioLegend^®^, 305,118, 1 µg/ mL), anti-CD11b-BV785^TM^ (BioLegend^®^, 301,346, 1.25 µg/ mL) and anti-CD62L-BV605 ^TM^ (BioLegend^®^, 304,834, 250 ng/ mL) and concentration-matched isotype controls (BioLegend^®^). The gating strategy included identification of neutrophil singlets using forward scatter – area (FSC-A) versus side scatter – area (SSC-A) plots, and FSC-A versus FSC- height (FSC-H) plots (5000–10,000 neutrophil events were analyzed). Median fluorescence intensity and percentage of positive cells for each marker were measured, the latter gated using isotype controls. Gating strategy demonstrated in Supplementary Figure 1. Compensation analyses were performed on FlowJo_v10.9.0 Software.

### Measurement of ex vivo Nucleic Acid Release

Neutrophils (2 x 10^5^ in assay media) were treated for 4.5 hours with 1 ng/ mL TGFβ1 or vehicle at 37° C/5% CO_2_. Cells were then fixed in 2% paraformaldehyde for 10 minutes at 37° C and centrifuged at 2200 x g for 10 minutes at 4° C. DNA contents of 100 µL cell-free supernatant was measured by incubating with 1 µM SYTOX^TM^ Green (Invitrogen) for 10 minutes at RT, and fluorescence measured using a BioTek Synergy 2 Fluorometric plate reader; excitation 485nm/emission 528nm.

### Assessment of TGFβ Receptor 1 Expression in Human Neutrophils

TGFβ receptor 1 (TGFβR1) expression was assessed by Western blotting as described in the Supplementary Methods. Primary antibodies used were rat anti-human TGFβR1/ALK-5 antibody (R&D systems, MAB5871, 1 µg/ mL) and rabbit anti-human GAPDH antibody (Abcam, ab181602).

### Phosphoflow Cytometric Analysis of pSmad2/3 to Explore Signaling Pathway

Phosphorylated Smad2/3 (pSmad2/3) expression was examined using Phosphoflow cytometry as described in the Supplementary Methods, with neutrophils incubated in assay media supplemented with 10 ng/ mL TGFβ1 or vehicle for 5–60 min. Primary antibodies used were anti-Smad2 (pS465/ pS467)/Smad3 (pS423/ pS425) PE conjugated antibody (BD Biosciences, 562586, 80 ng/ mL) or isotype control antibody (BioLegend, 400112).

### Statistical Analysis

Statistical analyses were performed using GraphPad Prism^®^ (GraphPad Software Ltd, San Diego, CA, USA). A Shapiro–Wilk test was performed to assess data distribution. Paired *T*-tests and Wilcoxon tests were used to assess differences between matched paired samples for normally distributed and skewed data, respectively. One-way ANOVA or Kruskal–Wallis tests were performed to compare differences between three or more independent groups that contained normally distributed or skewed data, respectively. Dunnett’s or Dunn’s post-hoc multiple comparisons tests were subsequently performed for normally distributed and skewed data, respectively. Data are presented as mean ± SE or median ± interquartile range (IQR). Statistical significance was determined by p-value < 0.05. “N” in figures refers to the number of biological replicates. All data from biological replicates is representative of a minimum of 2 technical replicates. Each figure should be regarded as an independent set of experiments.

## Results

### TGFβ1 Increases Neutrophil Migration Across Transwell Membranes

The initial effect of TGFβ1 on neutrophil movement was investigated using the transwell migration assay. Figure 2 shows that 0.1, 1 and 10 ng/ mL TGFβ1 significantly increased neutrophil migration across the transwell membrane (0.1 ng/ mL TGFβ1 mean fold change (FC) versus vehicle = 1.33 ±0.09; p<0.05: 1 ng/ mL TGFβ1 mean FC versus vehicle = 1.38 ±0.05; p=0.002: 10 ng/ mL TGFβ1 mean FC versus vehicle = 1.26 ±0.07; p<0.05).

**Figure 2:**
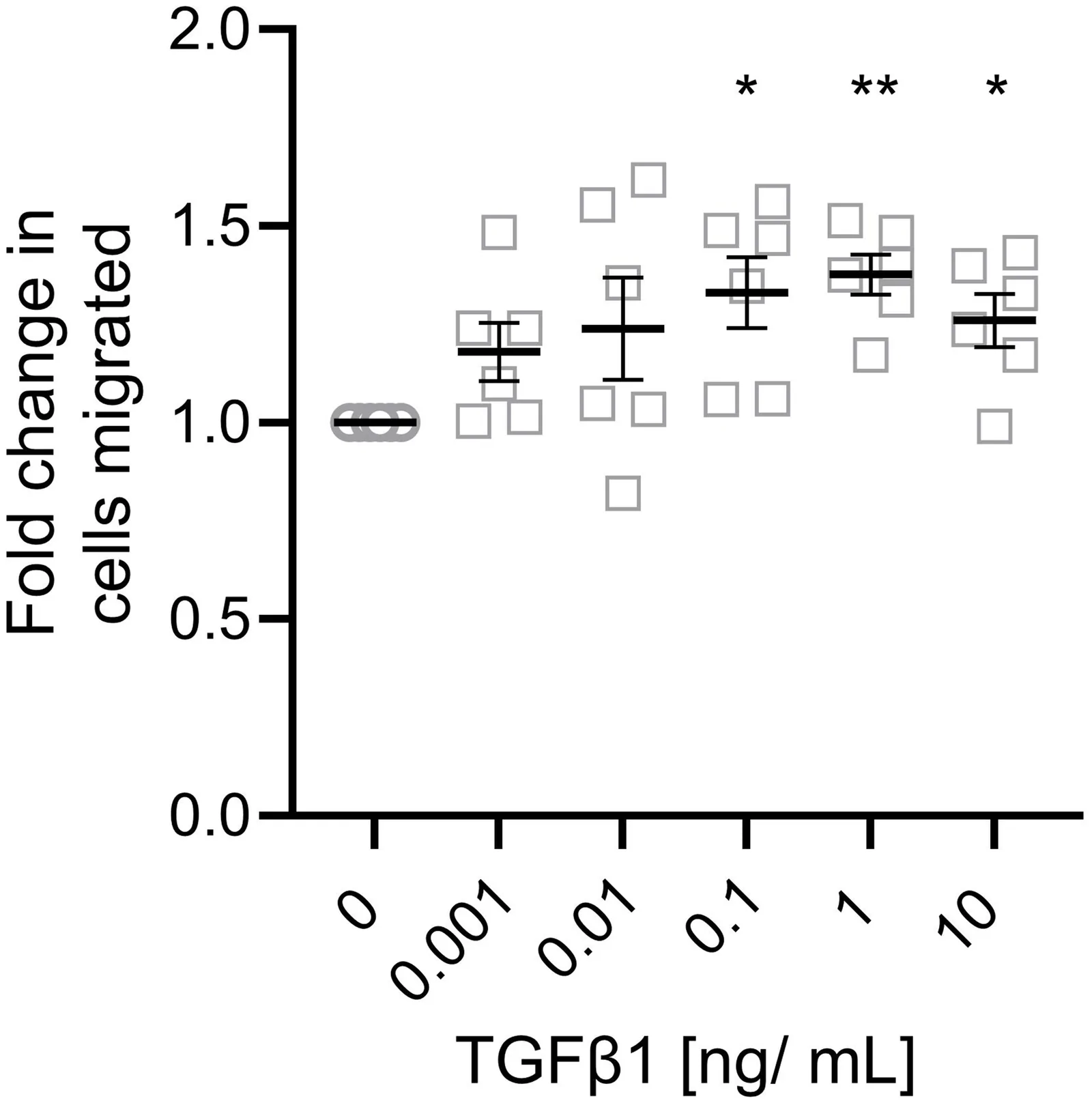
TGFβ1 increases neutrophil migration across transwell membranes. The number of neutrophils from healthy donors (n = 6) that migrated through polycarbonate transwell inserts into the bottom well containing vehicle or 0.001–10 ng/ mL TGFβ1 was counted by flow cytometry. *p<0.05, **p=0.002 compared to vehicle (one-way ANOVA and Dunnett’s multiple comparisons test). Bars are mean ±SE.

### TGFβ1 Increases Neutrophil Chemokinesis, but Not Chemotaxis

As the transwell assays do not discriminate directed migration from increased random motility we employed the Insall chamber assay to differentiate between direction-dependent (chemotaxis) or direction-independent movement (chemokinesis).^33^ The direction-independent parameters were significantly increased in response to 10 ng/ mL TGFβ1 compared to vehicle (speed = 4.89 ±0.56 µm/ min versus 2.17 ±0.25 µm/ min, distance travelled = 55.41 ±6.32 µm versus 24.53 ±2.78 µm, and displacement = 21.05 ±2.98 µm versus 7.84 ±1.33 µm; p<0.001, *n* = 7) (Figure 3A–C).^22^ However, TGFβ1 did not affect the direction dependent parameters (velocity and chemotactic index) (*n* = 7) (Figure 3D and E). A higher concentration of 30 ng/ mL TGFβ1 was also examined within the Insall chamber (Supplementary Figure 2) with results revealing the speed and distance travelled by neutrophils was increased when compared to vehicle control (p<0.05, *n* = 7).

**Figure 3:**
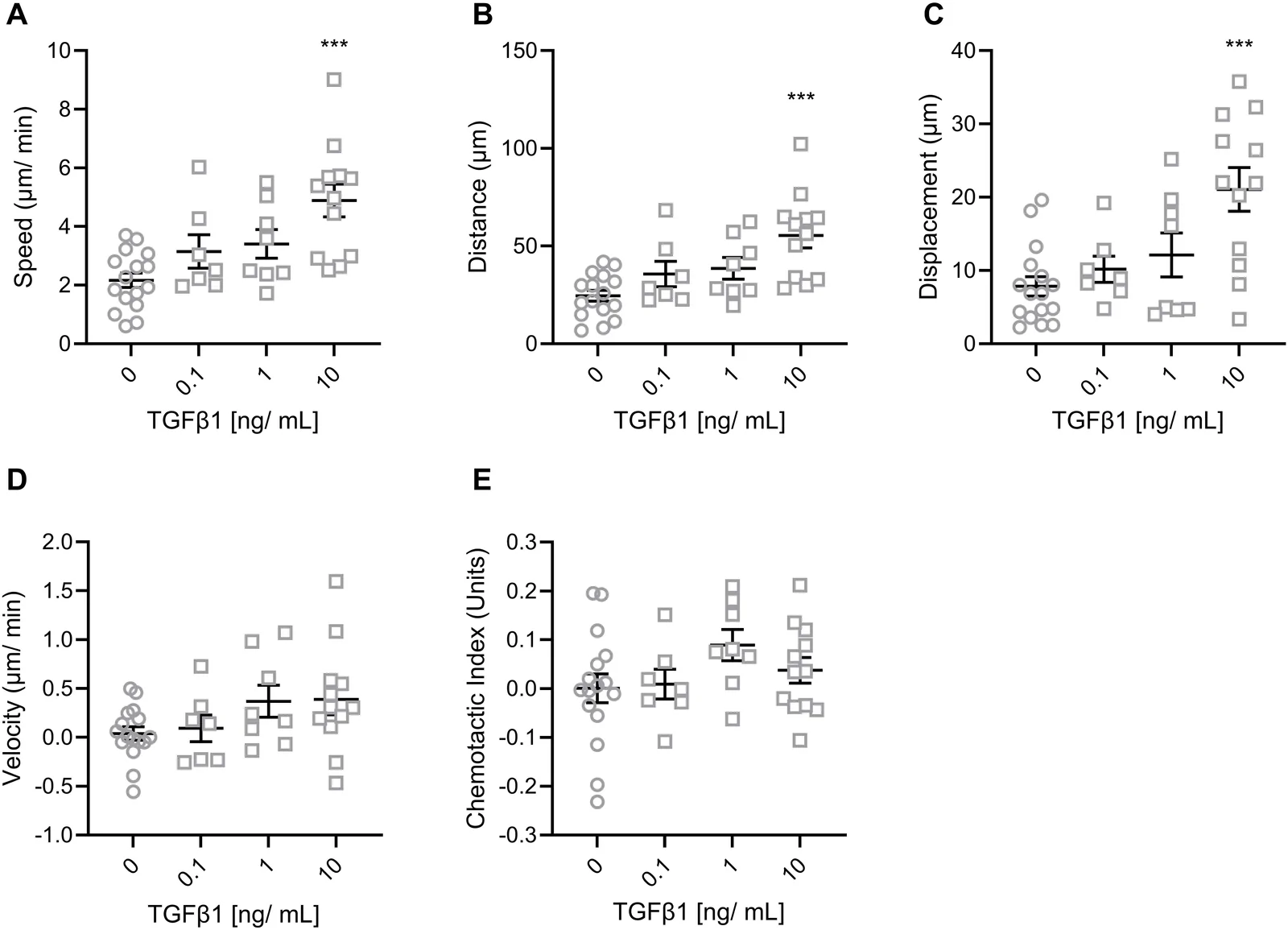
TGFβ1 increases neutrophil chemokinesis, not chemotaxis. Neutrophils isolated from healthy donors (*n* = 7) were added to the Insall chamber.^33^ TGFβ1 (0.1–10 ng/ mL) or vehicle was added to the chemoattractant well and neutrophil movement was tracked in real-time over 12 minutes. Mean ±SE displayed. *** p<0.001 compared to vehicle ((**A** and **B, D** and **E**): One-way ANOVA and Dunnett’s multiple comparisons tests. (**C**) Kruskal–Wallis and Dunn’s multiple comparisons tests). Data from individual donors is shown by the open symbols.

### TGFβ1 Pre-Treatment Enhances Selected Neutrophil Movement Responses to IL-8 and fMLP

Pre-treatment with 10 ng/ mL TGFβ1 increased the speed and distance travelled by neutrophils in response to 100 nM IL-8 (Figure 4A) and velocity in response to 10 nM fMLP (Figure 4B).

**Figure 4:**
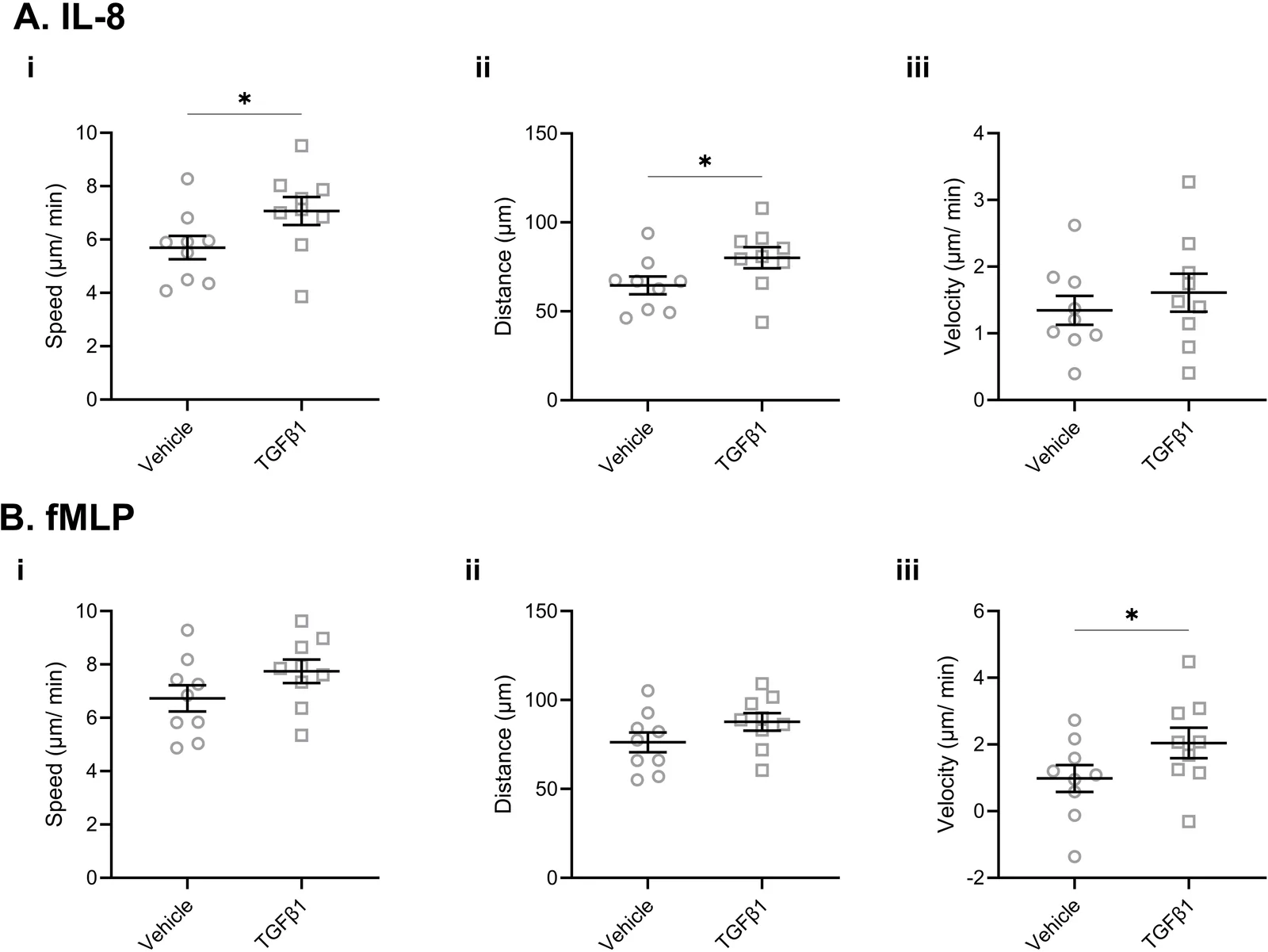
TGFβ1 pre-treatment amplifies movement responses to chemoattractants. Neutrophils isolated from healthy donors (*n* = 9) were cultured for 15 minutes in the presence of vehicle or 10 ng/ mL TGFβ1. Neutrophils were then washed and added to the Insall chamber containing either 100 nM IL-8 (**A**) or 10 nM fMLP (**B**). Neutrophil speed (i), distance travelled (ii) and velocity (iii) were measured over 12 minutes. Mean ±SE bar lines are displayed. * p<0.05 compared to vehicle (paired *T*-tests). Data from individual donors is shown by the open symbols.

### TGFβ1 Increases Extracellular Superoxide Anion Production

Pre-treatment of neutrophils with 10ng /mL TGFβ1 increased total extracellular superoxide anion associated chemiluminescence (FC versus vehicle = 1.41 ±0.16; p<0.05, *n* = 6, Figure 5).

**Figure 5:**
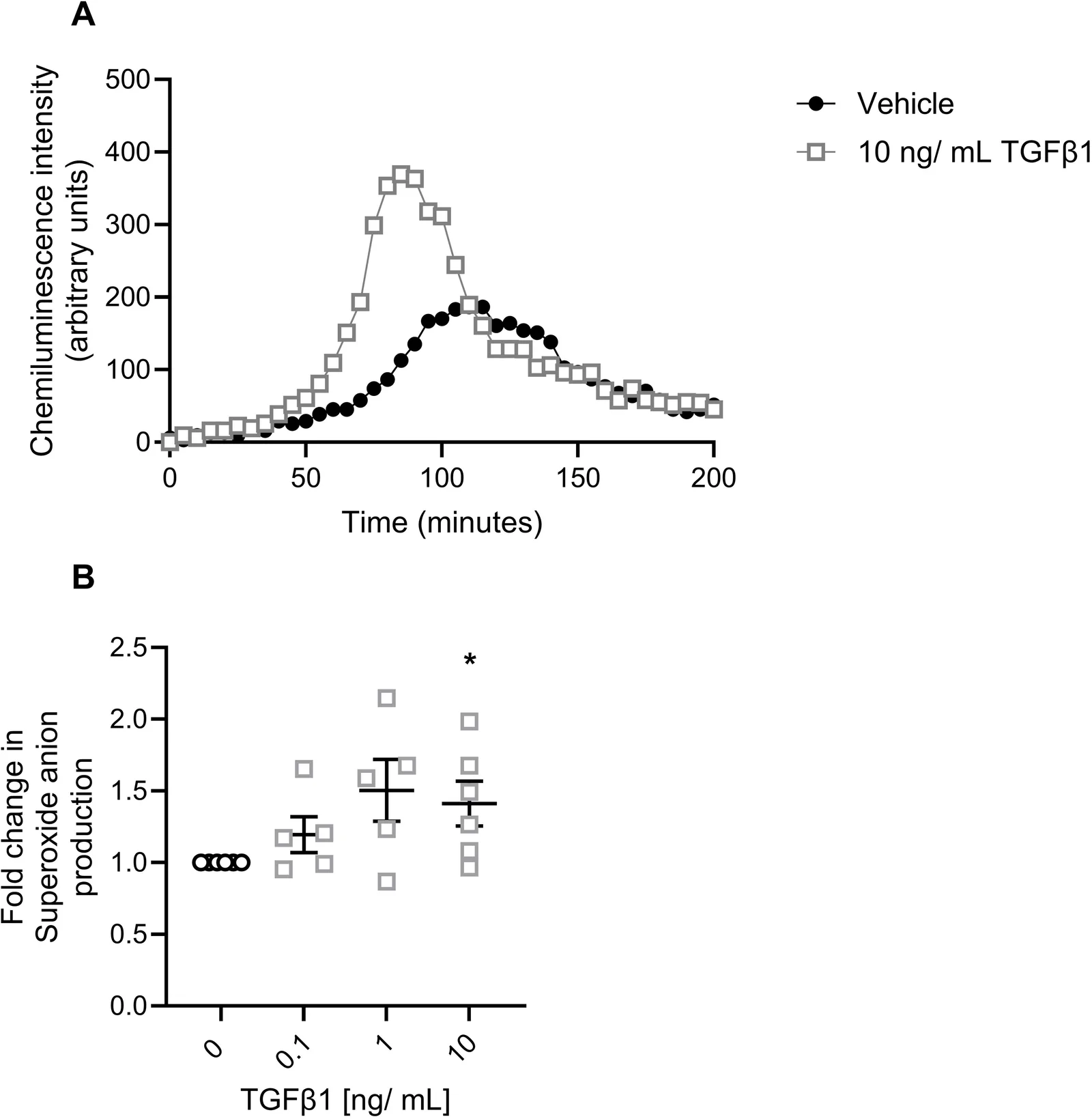
TGFβ1 increases superoxide anion production. The effect of 0.1–10 ng/ mL TGFβ1 on extracellular superoxide anion production was assessed using the lucigenin-based chemiluminescence assay. Total superoxide anion production was determined by measuring the area under the curve. (**A**) Representative plot of superoxide anion production, presented as chemiluminescence intensity values after exposure with vehicle (black circles/line) or 10 ng/ mL TGFβ1 (grey squares/line). (**B**) Mean ±SE fold change in total superoxide anion production compared to vehicle are displayed (0 and 10 ng/ mL TGFβ1; *n* = 6: 0.1 and 1 ng/ mL TGFβ1; *n* = 5). Individual donor data points are shown. * p<0.05 compared to vehicle (paired *T*-test).

### TGFβ1 Increases Extracellular DNA Release from Neutrophils

To explore whether TGFβ1 might promote NET-associated responses, extracellular DNA release following 4.5 hour exposure to 1 ng/ mL TGFβ1 was measured. TGFβ1 increased extracellular DNA release compared with vehicle control (mean 5130 ±710 relative fluorescence units (RFU) versus mean 4393 ±631 RFU; p<0.01, *n* = 6, Figure 6). However, this was not accompanied by a detectable increase in NET-area when measured by immunofluorescence microscopy (SYTOX_TM_ Green stained nucleic acid) (Supplementary Figure 3). Annexin V/ propidium iodide (PI) assays (Supplementary Methods) demonstrated that neutrophil viability was unaffected by TGFβ1 exposure (Supplementary Figure 4).

**Figure 6:**
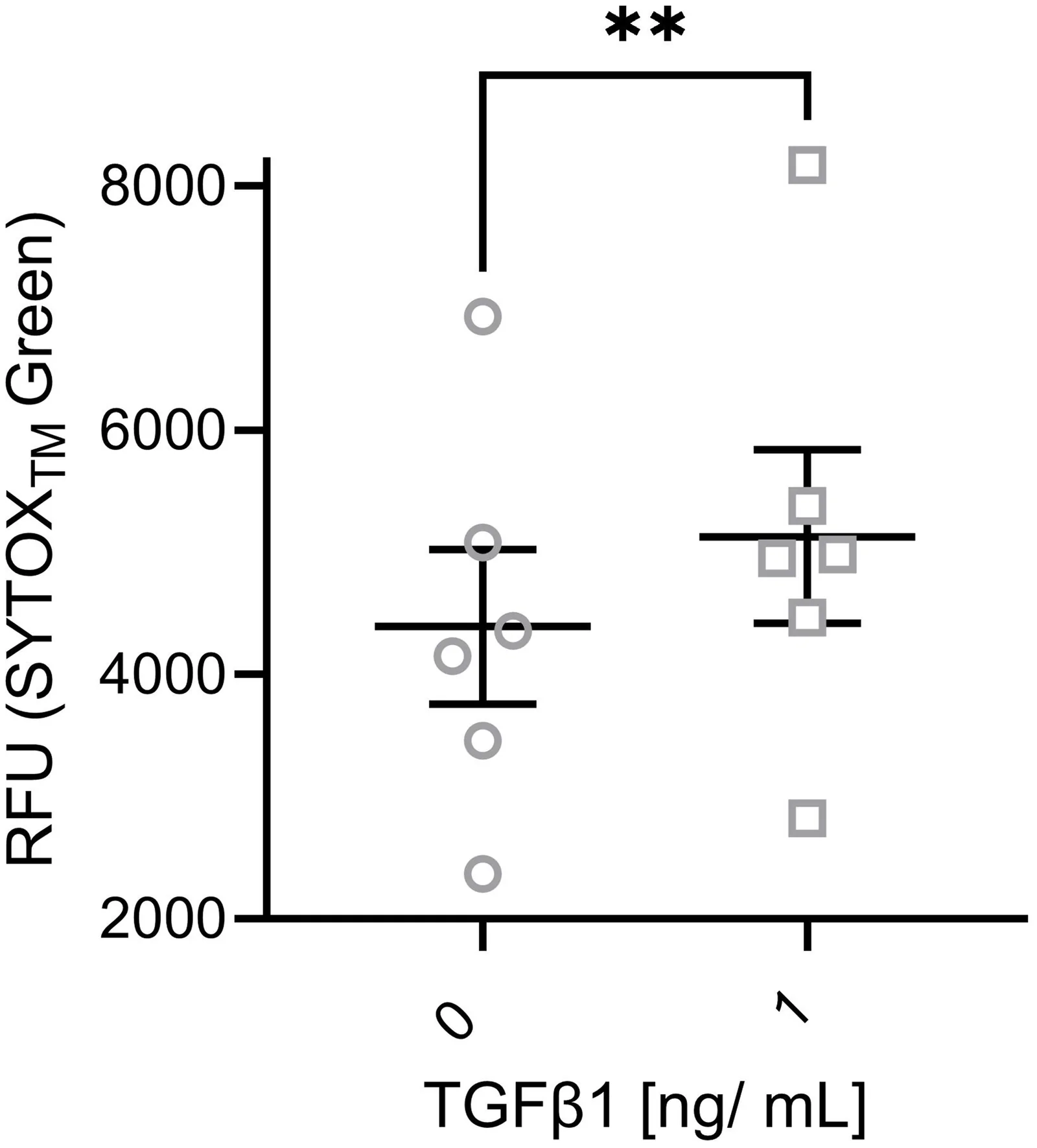
TGFβ1 increased extracellular DNA release from neutrophils. The effect of 4.5 hour exposure of 1 ng/ mL TGFβ1 on extracellular DNA release was assessed by fluorometry. Mean ±SE relative fluorescence units (RFU) of SYTOX_TM_ Green are displayed (*n* = 6). Data point per donor. ** p<0.01 when compared to vehicle (paired *T*-test).

### TGFβ1 Does Not Affect the Cell Surface Expression of CD11b, CD62L or CD66b

To examine whether TGFβ1 impacted upon the activation status of neutrophils, its effects on the cell surface expression of the adhesion markers, CD11b and CD62L, and the degranulation indicator CD66b, were assessed. Exposure to 100 nM IL-8 (positive control) increased the expression of CD11b and CD66b but 10 ng/ mL TGFβ1 did not affect the expression of these markers over 5 to 60 minutes exposure (Supplementary Figure 5). This again suggests selective rather than a global priming-like phenotype.

### TGFβ1 Signaling in Human Neutrophils

These observations imply a direct functional effect of TGFβ1 on neutrophils. We sought supportive evidence of TGFβ1 responsiveness in these cells. Western blotting of neutrophil lysates from 3 healthy donors identified a band at the expected molecular weight for TGFβR1, 53kDa (Figure 7A), consistent with expression of this receptor in mature neutrophils. Furthermore, TGFβR1 blockade attenuated extracellular DNA release from neutrophils exposed to 10 ng/ mL TGFβ1 (Supplementary Figure 6). Together this supports that this cytokine signals through this receptor in human neutrophils. To explore whether TGFβ1 exposure engages canonical downstream signaling pathways, phosphorylated Smad2/3 was assessed. Given the rapid dynamics of Smad phosphorylation, cytometric analysis was used. Smad2/3 phosphorylation was significantly elevated at 5 and 60 minutes after exposure to 10 ng/ mL TGFβ1 (Figure 7B). This supports the capacity of human neutrophils to respond to TGFβ1 through the canonical Smad-dependent signaling pathway.

**Figure 7:**
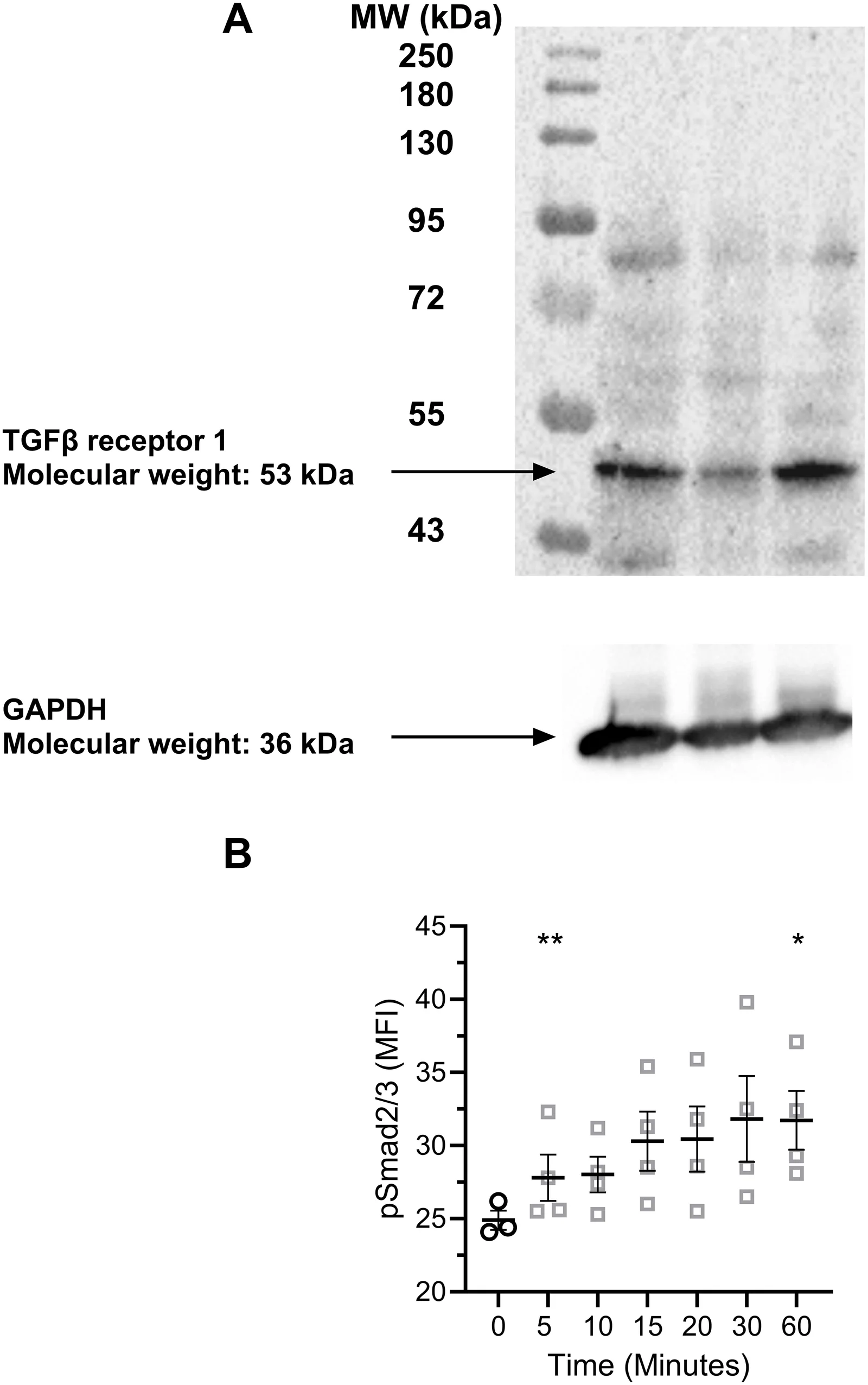
TGFβ1 signaling in human neutrophils. (**A**) Lysates were prepared from neutrophils isolated from three healthy donors. TGFβ receptor 1 (TGFβR1) and GAPDH expression were assessed sequentially on the same experimental blot. (**B**) Neutrophils isolated from healthy donors (*n* = 4) were cultured for 5 to 60 minutes in the presence of 10 ng/ mL TGFβ1. Timepoint 0 represents vehicle exposed neutrophils cultured for 60 minutes (*n* = 3). pSmad2/3 expression, represented as median fluorescence intensity (MFI), was assessed by flow cytometry. Histograms are mean ±SE. * p=0.02, ** p=0.007 compared to vehicle (paired *T*-tests).

## Discussion

In this study, conventional transwell assays suggested that TGFβ1 enhances neutrophil migration, consistent with published literature studies.^17^,^20^,^29^,^36^ However, direct live-cell tracking demonstrated this effect reflects increased chemokinesis rather than chemotaxis. Additionally, TGFβ1 enhanced selected responses to canonical chemoattractants and increased extracellular superoxide anion release. Together this supports the interpretation that TGFβ1 promotes a priming-like functional state in mature human neutrophils.

Others have reported that TGFβ1 affects neutrophil movement using a variety of in vitro models, such as transwell/Boyden chamber assays or the under agarose method.^17^,^20^,^29^,^36^ Although the under agarose method quantifies movement towards the source of the chemoattractant, these other techniques provide less insight into directional movement; a key feature influencing recruitment to the source in the lung. The transwell method/Boyden chamber does not distinguish between chemotaxis and chemokinesis, with movement calculated by counting the number of neutrophils that have travelled through ± adhered to the polycarbonate filter.^20^,^22^ Therefore, an increase in neutrophil transmigration within these modeling systems is likely a secondary effect due to increased random locomotion that is dose dependent. Reibman et al reported that approximately 1 pg/ mL TGFβ1 elicited chemotaxis using the under agarose method, which was attenuated by TGFβ1 antagonism.^29^ However, the Insall chamber method used in this study, with real-time tracking of neutrophils in the presence of a chemoattractant gradient, provides clearer insight into chemotactic parameters versus chemokinetic ones.

In our study, neutrophils were exposed to a range of TGFβ1 concentrations, which aimed to reflect levels previously measured within the circulation and bronchoalveolar lavage fluid of patients with interstitial lung disease and other pathologies, such as lung cancer.^15^,^16^,^37^,^38^ Ten ng/ mL TGFβ1 increased neutrophil speed approximately 2.3 fold compared to vehicle control, but not at lower concentrations. However, 30 ng/ mL TGFβ1 increased neutrophil movement 1.4 fold. This is consistent with previous studies reporting a bell-shaped response to this cytokine.^20^ Our study does not confirm that TGFβ1 is a specific chemoattractant despite this increase in speed of movement, whereas 100 nM IL-8 (a physiologically recognized chemoattractant) significantly increases direction dependent and independent parameters.^39^

Exposure to a stimulus can result in quiescent neutrophils transitioning to a primed or activated state. Priming agents augment responsiveness to activating stimuli, but can also independently induce functional changes in neutrophils.^22^ For example, TNFα (an established priming agent) as well as amplifying chemotactic responses to fMLP in vitro, can (in isolation) drive neutrophil chemokinesis.^40^ In the current study, we noted that pre-treatment with TGFβ1, amplified movement responses to the established chemoattractants IL-8 and fMLP. We propose that the chemokinetic and superoxide responses induced by TGFβ1 are consistent with a priming-like phenotype. This was supported by the observation that TGFβ1 treatment increased total superoxide anion production, reflective of an increase in NADPH-oxidase mediated respiratory burst. Amplification of this process is seen with many priming cytokines, for example, TNFα increases superoxide production via phosphorylation of p47^phox^, a cytoplasmic component of NADPH oxidase.^41^ Our findings of TGFβ1 additive chemokinetic effects may explain Chong et al observations that IPF peripheral blood neutrophils have an amplified chemotactic response to fMLP compared to non-interstitial lung disease controls ex vivo.^42^

TGFβ signaling in human neutrophils remains poorly understood. In other cell types, TGFβ1 activity is reliant on binding to and subsequent formation of a heterocomplex of two cell membrane serine and threonine kinase receptors, TGFβR1 and TGFβR2.^43^ Downstream signaling then ensues via canonical Smad-dependent and/or non-canonical Smad-independent pathways.^44^,^45^ Here, we showed that human neutrophils express TGFβR1 and that TGFβ1 appears to signal through this receptor. Furthermore, TGFβ1 increases phosphorylation of the canonical signaling molecules, Smad2/3. This is in keeping with the few studies to-date that have reported TGFβR1 expression in human neutrophils (detected by polymerase chain reaction^46^ and affinity binding studies^19^). In addition, it supports research by Hein et al that noted TGFβ1 increased expression of p-Smad3c in human neutrophils.^18^ Therefore, our receptor and Phosphoflow data support the interpretation that human neutrophils are directly TGFβ1 responsive and are capable of engaging the canonical Smad signaling pathway following TGFβ1 exposure. Further work using receptor blockade or kinase inhibition would be required to directly determine whether this pathway is linked to all the functional effects observed.

In the current study, TGFβ1 did not affect the cell surface expression of adhesion (CD11b and CD62L) or degranulation markers (CD66b), which can change on transitioning to a primed and/or activated state.^47–53^ This is in keeping with previous research that observed that TGFβ1 had no effect on degranulation.^29^ However, this does not exclude TGFβ1 mediated priming capabilities. For example, GM-CSF and TNFα are both considered priming agents despite CD66b being unaffected by GM-CSF, and TNFα not consistently increasing CD11b expression.^40^,^54^

TGFβ1 increased extracellular DNA release from neutrophils, but was not accompanied by a detectable increase in microscopy-based NET area. These findings indicate that TGFβ1 may influence extracellular DNA release under these conditions, but does not provide definitive evidence that TGFβ1 promotes NET formation. The imaging technique adopted here may not have been able to detect small amplitude NET formation. Neutrophil viability was maintained during cytokine exposure suggesting that TGFβ1 may induce a distinct type of NET formation called “vital” NETosis,^55^ however, this remains speculative. The process of and subsequent measurement of “vital” NETosis remains poorly understood. Given the methodological challenges of separating NETosis from other sources of extracellular DNA, this requires further investigation to fully define this effect.

As demonstrated here, research to date exploring the effects of TGFβ1 on human neutrophils has yielded conflicting results. There are many potential reasons for this. Variability between in vitro models of neutrophil movement has already been described, other factors are proposed here. Human neutrophils are difficult to examine ex vivo due to high susceptibility to activation influenced by numerous factors including isolation technique and processing time.^56^ In the current study, neutrophils were manipulated with the utmost care, which included gentle handling and minimizing processing times. Neutrophils were isolated using dextran sedimentation and discontinuous Percoll^®^ density gradient centrifugation.^32^ This technique has been shown to yield equivalent results to neutrophils examined in whole blood, although others question this validation.^56^,^57^ Lack of consensus on the optimum method of neutrophil isolation has thus likely resulted in heterogeneity between studies. TGFβ1 bioactivity and interdonor responsiveness in vitro may also account for interstudy variability. TGFβ1 bioactivity is reliant on activation and the current study was performed by acidification using 4 mM hydrochloric acid as per manufacturer’s instructions for consistency. Variability in the biological activity of TGFβ1 used in other studies could also lead to variable outcomes in the literature, with others not describing the supplier or method of activating the recombinant human TGFβ1.^17^,^18^,^21^ As human neutrophils are primary cells and have a sensitive nature, likely greater sample sizes are needed to yield appropriate statistical power. We used greater sample sizes than previous studies^17^,^18^ to provide sufficient statistical power. A final consideration related to TGFβ1 responsiveness is the potential presence of polymorphic sequence changes in TGFβR1 that have been identified elsewhere, although not yet identified in neutrophils.^58^

Future work is needed to build on the current data to determine the mechanism by which TGFβ1 may be promoting neutrophil chemokinesis and its complete priming and/or activation capabilities. Models that have greater physiological complexity, for example, in the context of pulmonary fibrosis including components that represent passage from the vasculature into the alveolar space, such as endothelial cells, extracellular matrix and epithelial cells would provide more physiological data. Furthermore, given the importance of TGFβ1 in diseases such as IPF, assessment of whether neutrophils isolated from patients with such conditions display a primed phenotype and/or respond in the same way to this pro-fibrotic cytokine would be essential.

## Conclusions

In summary, we have shown that TGFβ1 promotes human neutrophil chemokinesis, rather than chemotaxis, and our findings suggest that TGFβ1 induces a selective priming-like state with potential relevance to neutrophil behavior in conditions such as fibrotic lung disease.

## Data Availability

All data is stored on a University encrypted database. The datasets are not publicly available and can solely be accessed by the authors. However, data used and/or analyzed during the current study are available from the corresponding author on reasonable request.

## Abbreviations

AUC, Area under the curve; DPBS, Dulbecco’s Phosphate Buffered Saline; FC, Fold change; fMLP, N-Formylmethionine-leucyl-phenylalanine; FSC-A, Forward scatter - area; FSC-H, Forward scatter - height; HRP, Horseradish peroxidase; IPF, Idiopathic pulmonary fibrosis; MFI, Median fluorescence intensity; NET, Neutrophil extracellular trap; PI, Propidium iodide; PMA, Phorbol-12-myristate-13-acetate; RT, Room temperature; SSC - area, Side scatter - area; SDS, Sodium dodecyl sulphate; TBST, Tris-buffered saline containing 3% Tween^®^ 20; TGFβ1, Transforming growth factor–beta 1; TGFβR1, TGFβ receptor 1.
